# The Different Ways Multi-Strain Probiotics with Different Ratios of *Bifidobacterium* and *Lactobacillus* Relieve Constipation Induced by Loperamide in Mice

**DOI:** 10.3390/nu15194230

**Published:** 2023-09-30

**Authors:** Chenyue Zhang, Linlin Wang, Xiaoming Liu, Gang Wang, Xinmei Guo, Xuecong Liu, Jianxin Zhao, Wei Chen

**Affiliations:** 1 State Key Laboratory of Food Science and Resources, Jiangnan University, Wuxi 214122, China; 7210112071@stu.jiangnan.edu.cn (C.Z.); liuxm@jiangnan.edu.cn (X.L.); wanggang@jiangnan.edu.cn (G.W.); zhaojianxin@jiangnan.edu.cn (J.Z.); weichen@jiangnan.edu.cn (W.C.); 2 School of Food Science and Technology, Jiangnan University, Wuxi 214122, China; 3 (Yangzhou) Institute of Food Biotechnology, Jiangnan University, Yangzhou 225004, China; 4 JinQiao Biotechnology Co., Ltd., Huai’an 223010, China; xinmei@bioflag.com (X.G.); xuecong.liu@bioflag.com (X.L.); 5 National Engineering Research Center for Functional Food, Jiangnan University, Wuxi 214122, China

**Keywords:** probiotic supplements, short-chain fatty acids, gastrointestinal regulatory transmitters, constipation

## Abstract

Constipation is currently one of the most common gastrointestinal disorders, and its causes are diverse. Multi-strain probiotics are often considered a more effective treatment than single-strain probiotics. In this study, a constipation model was constructed using loperamide hydrochloride to evaluate the ability of a multi-strain probiotic combination of four different ratios of *Bifidobacterium* and *Lactobacillus* to regulate intestinal flora, relieve constipation, and explore the initial mechanism in mice. After four weeks of probiotic intervention, BM1, BM2, and PB2 effectively relieved constipation; however, the pathways involved were different. The *Bifidobacteria*-dominated formulations BM1 and BM2 mainly changed the composition and structure of the intestinal flora and significantly decreased the relative abundance of *Tyzzerella*, *Enterorhabdus*, *Faecalibaculum*, *Gordonibacter*, and *Mucispirillum* in stool; increased the relative abundance of *Parabacteroides* and the content of short-chain fatty acids (SCFAs) in stool; restored motilin (MTL) and vasoactive intestinal peptide (VIP) levels; and downregulated interleukin 6 (IL-6) and IL-8 levels in serum. This repaired the inflammatory response caused by constipation. Finally, it promoted peristalsis of the gastrointestinal tract, increasing stool water content, and relieving constipation. While *Lactobacillus*-dominated formula PB2 mainly restored the levels of serum neurotransmitters (MTL, SP (substance P), VIP and PYY (Peptide YY)) and inflammatory factors (IL-1, IL-6 and IL-8), it significantly decreased the relative abundance of *Tyzzerella*, *Enterorhabdus*, *Faecalibaculum*, *Gordonibacter* and *Mucispirillum* in stool; it then increased acetic acid content, thereby reducing the level of inflammation and changing stool properties and gastrointestinal motility.

## 1. Introduction

Chronic constipation can be divided into functional constipation and constipation-type irritable bowel syndrome [1], which is a dysfunctional gastrointestinal tract disease [2,3]. In addition to symptoms such as difficulty in defecation, a low frequency of defecation, and dry and hard stools [4], intestinal microecological disorders also occur [3,5]. Existing drugs have side effects [6]; for example, prucalopride can effectively promote colonic transport and improve intestinal function, but there are still adverse reactions such as headache, nausea, abdominal pain, and diarrhoea [7]; lubiprostone can activate specific chloride channels in intestinal epithelial cells and increase intestinal water secretion, but it is not absorbed by the whole body, and side effects such as nausea may occur [8]. Therefore, making nontoxic, non-dependent probiotic, prebiotic, synbiotic, and postbiotic interventions is a good choice [1,9].

Probiotics are live microorganisms that, when consumed in sufficient quantities, have a positive influence on host health [10]. There is a growing body of research on the effects of probiotics on constipation [11,12]. Some studies have shown that probiotics that relieve constipation symptoms are mostly associated with regulation of the expression of specific genes in the intestine [13], affecting the synthesis and secretion of neurotransmitters [14]. Some studies have also found that probiotics affect the intestinal environment and alter immune responses to specific antigens [15,16,17]. There is some variation in the effectiveness of different probiotic strains in relieving constipation, with some strains showing effectiveness and others being ineffective [18]. Studies have also reported that multi-strain probiotics are more effective than single-strain probiotics at improving symptoms of constipation [19]. In the study of Helicobacter pylori infection, MacPherson et al. [20] found that no significant improvement was found in the use of a single probiotic strain or a mixture of four strains, whereas the mixture of two strains significantly relieved the symptoms of the infection. Jeong et al. [21] gave multiple strains of probiotics to rats for 14 days. The results showed that the probiotics could relieve the constipation induced by loperamide. Multi-strain probiotic combinations have undoubtedly become a research hotspot [22,23]. In addition, there is much information about the improvement of multi-strain probiotics in non-alcoholic fatty liver disease [24], severe major depressive disorder, and accompanying gastrointestinal syndromes [25]. However, most studies have focused on whether compounded strains are effective, and few studies have been conducted to explain the rationale for strain compounding.

In this study, strains *Bifidobacterium infantis* BLI-02 (*B. infantis* BLI-02) [26], *Bifidobacterium animalis* BB-115 (*B. animalis* BB-115) [27], postbiotic PE0401 [28], *Lacticaseibacillus rhamnosus* MP108 (*L. rhamnosus* MP108) [29], and *Lacticaseibacillus paracasei* CCFM2711 (*L. paracasei* CCFM2711) [30]), which showed good probiotic effects in previous animal experiments and clinical trials, were selected and combined (the formulations were: BM1; BM2: BM1 + CCFM2711; PB1: BM1 + PE0401; PB2: PB1 + CCFM2711). Furthermore, animal experiments were conducted to investigate whether probiotic combinations with different ratios of *Bifidobacterium* and *Lactobacillus* regulated the intestinal flora and relieved constipation. To provide a foundation and technical support for the development of probiotic formulations to relieve constipation, we investigated whether constipation could be relieved from the perspective of gastrointestinal regulatory transmitters, intestinal inflammatory factors, and intestinal microecology.

## 2. Materials and Methods

### 2.1. Materials and Reagents

Loperamide hydrochloride capsules were purchased from Xi’an Janssen Pharmaceutical Company, Ltd. (Xi’an, China). Arabic gum powder and activated carbon powder were purchased from Sinopharm Chemical Reagent Company (Shanghai, China). Eosin-Methylene Blue Agar (EMB), Bile Esculin Azide Agar, BBL Agar, LBS Agar, and TSC Agar Base were purchased from Qingdao Hi-Tech Industrial Park Hopebio Biotechnology Co. (Qingdao, China). Mouse acetylcholine (Ach); mouse gastrin (Gas); mouse Peptide YY (PYY); mouse substance P (SP); mouse motilin (MTL); mouse 5-hydroxytryptamine (5-HT); mouse vasoactive intestinal peptide (VIP); mouse growth inhibitor (SS); mouse interleukin 1 (IL-1), 6 (IL-6), 8 (IL-8), and 10 (IL-10); and mouse transforming growth factor β (TGF-β) ELISA kits were purchased from Shanghai Enzyme Link Biotechnology Co. (Shanghai, China). The activated carbon solution was prepared according to the following method: 10 g of Arabic gum was added to 80 mL of water and was boiled until it became transparent; then, 5 g of activated carbon (in the form of powder) was weighed, added to the mixture, and boiled three times before being cooled and diluted with water to form 100 mL [31]. This solution was stored at 4 °C in the refrigerator and was shaken well before use.

### 2.2. Probiotics

All four probiotic supplement lyophilized powders were provided by Bioflag Biotech Co., Ltd. (Tainan, Taiwan)—namely BM1, BM2, PB1, and PB2 probiotic supplements with the specific strain formulations shown in Table 1 and lyophilized with maltodextrin as an excipient. Among these, *L. rhamnosus* MP108 was isolated from the intestines of healthy infants. *B. infantis* BLI-02 was isolated from the milk of healthy humans. *B. animalis* BB-115 was isolated from the human intestine. *L. paracasei* CCFM2711 was obtained from the Strain Bank of the Food Biotechnology Center of Jiangnan University (Wuxi, China); Postbiotic PE0401 was obtained from Bioflag Biotech Co., Ltd. (Tainan, Taiwan). They were stored in the freezer at −20 °C before use, the powder was removed on the day of gavage, and a volume of sterile saline was added to achieve a density of 4.1 × 10^8^ CFU/mL. The solution was mixed well and placed on ice.

### 2.3. Animals and Experimental Design

A total of 70 6-week-old specific pathogen-free (SPF)-grade C57BL/6J male mice were purchased from GemPharmatech LLC. (Nanjing, China). The methods and procedures used in this animal experiment were performed in accordance with the Guidelines for the Care and Use of Laboratory Animals of Jiangnan University (SYXK 2021-0056), reviewed and approved by the Animal Ethics Committee of Experimental Animals of Jiangnan University (Qualified No. JN. No 20211215c1200515[531]), and in accordance with the European Union Guide for Laboratory Animals (Directive 2010/63/EU). During the experimental period, all mice were fed with food and water ad libitum and fed in IVC cage boxes. Mice were fed standard chow during the experimental period and housed under the following environmental conditions: temperature 25 ± 2 °C, relative humidity 50 ± 5%, 12 h of light, and 12 h of darkness. Animal experiments were initiated one week after acclimatization.

**Figure 1:**
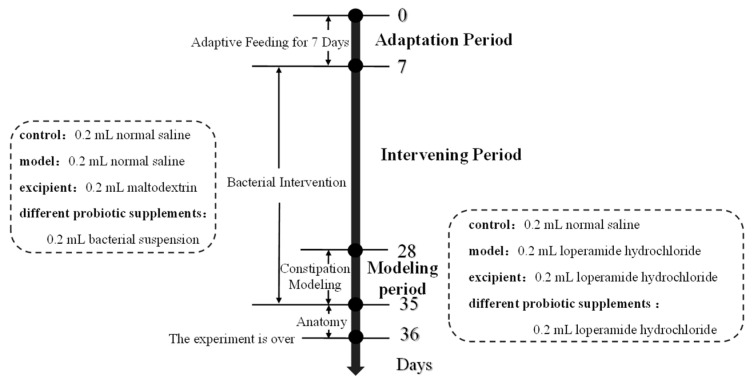
The schedule of the animal experiment.

The 70 mice were randomly divided into seven groups (10 mice per group): a blank control group (control), model group (model), adjuvant group (excipient), and four probiotic supplementation intervention groups. On days 7–28, the blank control and model groups were gavaged with sterile saline; the adjuvant group with maltodextrin solution resuspended in sterile saline; and the bacterial group with BM1, BM2, PB1, and PB2 resuspended in sterile saline at a dose of 0.2 mL of 4.1 × 10^8^ CFU/mL. On days 29–35, the other groups were gavaged with 15 mg/(kg·bw) loperamide hydrochloride for 1 h, whereas the blank control group received sterile saline. After 1 h, the model group was gavaged with sterile saline and all groups except the control continued to be fed 0.2 mL of the corresponding bacterial suspension or sterile saline. The experimental groups and procedures are presented in Table 2 and Figure 1.

### 2.4. The Collection of Mouse Tissues and Serum

Mouse faeces were collected weekly and stored in a -80 °C freezer for backup as soon as possible. The mice were fasted overnight after the experiment. They were intraperitoneally administered 100 mg/(kg·bw) of ketamine the next day, and after the completion of anaesthesia, blood was extracted from the eyes, and the mice were sacrificed via cervical dissection. Sterilized Eppendorf tubes measuring 1.5 mL were used to collect blood samples, which were left for 1 h and centrifuged at 3000× *g* for 15 min; the supernatant was divided and frozen at −80 °C for further analysis. The mice were dissected, the whole intestine (stomach to rectum) was removed for photography, and the rest of the tissues were immediately placed in liquid nitrogen and stored in a −80 °C freezer.

### 2.5. Experiment on Regulating the Function of Intestinal Flora

The experimental design and determination of relevant indexes were carried out with reference to the “Test method for regulating intestinal flora” in the “Health Food Function Test and Evaluation Method (2022 Edition)” [32]. Two to three faecal samples were aseptically collected from the anus of the mice on days 0 and 28 of the intervention and placed in sterile centrifuge tubes. The intestinal flora were detected according to the following assay: first, the wet weight of the stool was quantified, and then a 10-fold series of dilutions were performed; dilutions with colony counts in the range of 30–300 were selected and inoculated on the corresponding media for incubation. *Bifidobacteria* were selectively cultured in BBL Agar medium, *Lactobacillus* was selectively cultured in Lbs Agar medium, *Enterobacter* was selectively cultured in eosin-methylene blue Agar medium, *Enterococcus* was selectively cultured in Bile Esculin Azide Agar medium, and *Clostridium perfringens* was selectively cultured in TSC Agar medium. The colonies were counted according to the identification of colony morphology, Gram staining microscopy, biochemical reactions, etc. The number of *Bifidobacterium*, *Lactobacillus*, *Enterobacter*, *Enterococcus*, and *Clostridium perfringens* in each gram of wet stool was calculated. The results were determined using the logarithm (lg (CFU/g)).

### 2.6. Gastrointestinal Indices

#### 2.6.1. Faecal Water Content

Mouse faeces were collected weekly for faecal water content assays. The method was as follows: the mice were individually placed in clean cage boxes lined with filter paper after gavage. Fresh faeces were collected in 0.5 mL sterile Eppendorf tubes, weighed to obtain the wet weight, then freeze-dried and measured for the dry weight. The faecal water content was calculated according to Equation (1):

(1) $$\mathrm{Faecal}\;\mathrm{water}\;\mathrm{content}\left(\%\right)=\frac{\mathrm{wet}\;\mathrm{weight}\;\mathrm{of}\;\mathrm{the}\;\mathrm{faeces}\left(g\right)-\mathrm{dry}\;\mathrm{weight}\;\mathrm{of}\;\mathrm{the}\;\mathrm{faeces}\left(g\right)}{\mathrm{wet}\;\mathrm{weight}\;\mathrm{of}\;\mathrm{the}\;\mathrm{faeces}\left(g\right)}\times 100\%$$

#### 2.6.2. The Time of the First Black Stool Defecation

On the 35th day of the experiment, 0.2 mL of a mixture of loperamide hydrochloride and activated charcoal was gavaged, and the time between the gavage of the ink to the time when each mouse passed its first black stool was recorded as the time of the first black stool [33]. The treatment group that exceeded the time of the first black stool of the last mouse in the model group was considered ineffective, and the comparison of each treatment group with the model group was used to illustrate the differences among the groups in terms of constipation relief.

#### 2.6.3. Small Intestine Propulsion Rate

Mice were fasted without water for 12 h before dissection, and on day 36, all groups of mice except the control group were gavaged with 0.2 mL of a mixture of loperamide hydrochloride and activated charcoal. The control group was gavaged with a mixture of sterile saline and activated charcoal, and the mice were sacrificed 30 min later. The intestinal canal from the pylorus to the cecum was cut after opening the abdominal cavity of the mice, and this canal was pulled into a straight line; the length of the canal was measured as the “total length of small intestine”. The length from the pylorus to the ink front was the “front advancement length of activated charcoal solution” [34], and the small intestine advancement rate was calculated according to Equation (2):

(2) $$\mathrm{Small}\;\mathrm{intestine}\;\mathrm{propulsion}\;\mathrm{rate}\left(\%\right)=\frac{\mathrm{distance}\;\mathrm{propelled}\;\mathrm{by}\;\mathrm{the}\;\mathrm{activated}\;\mathrm{carbon}\left(\mathrm{cm}\right)\;}{\mathrm{total}\;\mathrm{length}\;\mathrm{of}\;\mathrm{the}\;\mathrm{small}\;\mathrm{intestine}\left(\mathrm{cm}\right)}\times 100\%$$

### 2.7. Biochemical Analyses

The following levels of constipation-related gastrointestinal regulatory transmitters were measured in mouse serum (50 μL) using ELISA kits: mouse motilin (MTL); gastrin (GAS); substance p (SP); vasoactive intestinal peptide (VIP); somatostatin (SS); Peptide YY (PYY); acetylcholine (Ach); 5-hydroxytryptamine (5-HT); the inflammatory factors Interleukin-1, 6, 8, and 10 (IL-1, IL-6, IL-8, IL-10); and transforming growth factor β (TGF-β). The procedure above was performed according to instructions.

### 2.8. The Determination of SCFA (Short-Chain Fatty Acid) Contents in Faeces

The contents of acetic acid (AA), propionic acid (PA), butyric acid (BA), isobutyric acid (IBA), valeric acid (VA), and isovaleric acid (IVA) content in mouse faeces were measured by GC-MS using an Rtx-Wax column (column length: 30 m, inner diameter: 25 μm). Thirty minutes after weighing, 30 mg of mouse faeces was lyophilized and suspended in saturated sodium chloride (0.5 mL). After using a tissue homogenizer to completely blend the material, 20 μL of 10% sulfuric acid was added and shaken vigorously for 30 s. Each sample was then given 1 mL of ether, and the mixture was centrifuged at 4 °C with 12,000× *g* for 15 min. A sample of the upper ether phase was taken, combined with 0.3 g of sodium sulphate, and centrifuged for 15 min at 4 °C and 12,000× *g*. For GC-MS detection, the supernatant was moved to a gas phase vial. The GC-MS conditions were as follows: Helium served as the carrier gas, and the flow rate and injection volume were 2 mL/min and 1 μL. The temperature was raised from 20 °C to 140 °C at a rate of 7.5 °C/min, then held at 200 °C for 3 min. A full-wavelength scan was selected for the GC-MS detection [35]. Standard curves for different SCFAs were prepared using the external standard method, and the concentrations of SCFAs in the samples (μmol/g) were calculated based on the obtained standard curves.

### 2.9. 16S rDNA Sequencing and Bioinformatics Analysis

DNA was extracted from mouse faeces using the Fecal FastDNA Spin Kit (MP Biomedical, Catalogue no. 6570200) according to the manufacturer’s instructions and was then used as a template for the polymerase chain reaction amplification of the V3–V4 fragments of the bacteria. 341F and 806R were used as forward and reverse primers, respectively. After electrophoresis on a 1.5% agarose gel, the microbial genomic DNA was stained with a 4% nucleic acid dye. The DNA Gel/PCR Purification Miniprep Kit (BW-DC3511-01, BIOMIGA, San Diego, CA, USA) was used to recover and purify the PCR products in accordance with the manufacturer’s instructions. To generate DNA libraries, DNA samples were mixed at similar concentrations after measuring the concentration of the extracted DNA using a NanoDrop spectrophotometer. They were subsequently sent to a MiSeq sequencer for sequencing using a MiSeq kit (Illumina, San Diego, CA, USA), and data processing and bioinformatics analysis were carried out using the QIIME2 platform. Quality control of the downstream data was performed according to the method described by Wang et al. [36]. Visualization was performed using OmicStudio (https://www.omicstudio.cn/home, accessed on 22 July 2023).

### 2.10. Statistical Analyses

The statistical analyses and plotting of the experimental data were expressed as “mean ± standard deviation” and performed using GraphPad Prism 8.0 (GraphPad Inc., San Diego, CA, USA) and IBM SPSS Statistics 22.0 (IBM Corp., Armonk, NY, USA). One-way ANOVA was used to analyse the differences between each group, and Fisher’s least significant difference (LSD) test was used to compare the experimental and model groups. The differences were considered statistically significant at *p* < 0.05. Positive results were determined by referring to the “Test method to help regulate intestinal flora” in the “Health food function test and evaluation method (2022 edition)”.

## 3. Results

### 3.1. The Effect of Multi-Strain Probiotic Intervention on the Body Weight of Constipated Mice Was Not Significant

A constipation mouse model was constructed using loperamide hydrochloride, and the body weights of the constipated mice increased significantly (*p* < 0.05), gaining approximately 2.2 g. The body weight of mice in the BM2-treated group increased significantly (*p* < 0.05) after intervention with various multi-strain probiotics; however, there was no significant difference in the body weight of the other intervention groups before and after the intervention (Table 3, *n* = 10).

### 3.2. Four Multi-Strain Probiotics Increased the Number of Beneficial Bacteria in the Intestines of Constipated Mice and Regulated Their Intestinal Flora

**Figure 2:**
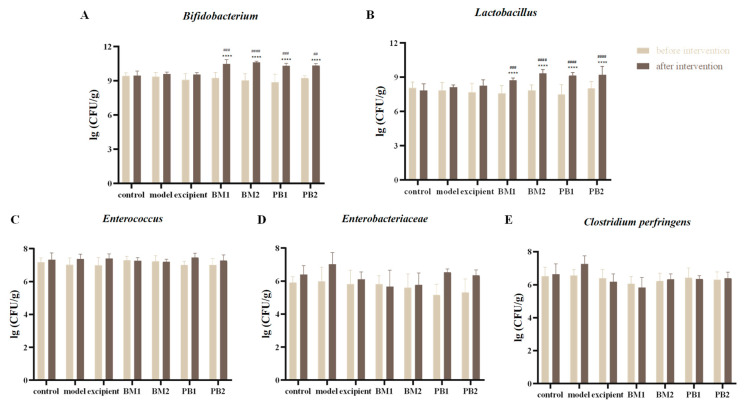
The effects of different probiotic supplements on the intestinal microflora of mice. (**A**) *Bifidobacterium*, (**B**) *Lactobacillus*, (**C**) *Enterococcus*, (**D**) *Enterobacteriaceae*, and (**E**) *Clostridium perfringens*. Data are shown as mean ± standard deviation. One-way ANOVA followed by Fisher’s LSD test for control and strain groups compared before and after the intervention, *n* = 10, **** *p* < 0.0001; and compared with the model group, ^##^ *p* < 0.01, ^###^ *p* < 0.001, ^####^ *p* < 0.0001.

The numbers of *Bifidobacterium*, *Lactobacillus*, *Enterobacter*, *Enterococcus*, and *Clostridium perfringens* in the intestinal flora of mice in the control and adjuvant groups did not differ significantly after 28 days of intervention (Figure 2A–E). After 28 days of the transoral administration of different multi-strain probiotics, the experimental group showed a significant increase (*p* < 0.0001) in the number of *Bifidobacterium* and *Lactobacillus* in the faeces of mice compared with that before the intervention (Figure 2A,B), and there were no significant changes in *Enterobacter*, *Enterococcus*, and *Clostridium perfringens* (Figure 2C–E). The comparison between the experimental and control groups after bacterial intervention showed the same results. The results are in line with those of the “Test method for regulating intestinal flora” in the “Health food function test and evaluation method (2022 edition)”.

### 3.3. Three Multi-Strain Probiotics Relieve Constipation

**Figure 3:**
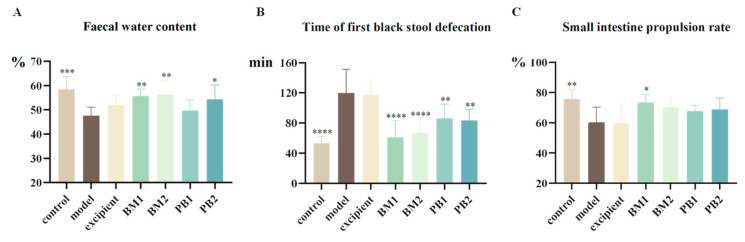
The effects of different probiotic supplements on gastrointestinal indicators in constipated mice. (**A**) Faecal water content, (**B**) Time of the first black stool defecation, and (**C**) Small intestine propulsion rate. Data are shown as mean ± standard deviation. One-way ANOVA followed by Fisher’s LSD test for the control and strain groups compared with the model group; *n* = 10, * *p* < 0.05, ** *p* < 0.01, *** *p* < 0.001, **** *p* < 0.0001.

After the application of loperamide hydrochloride treatment, Figure 3A shows that the faecal water content of the mice was significantly lower (*p* < 0.001), Figure 3B shows that the time it took them to pass their first black stool was significantly longer (*p* < 0.0001), and Figure 3C shows that the small intestinal propulsion rate was significantly slower (*p* < 0.01). Additionally, none of the gastrointestinal indices in the excipient group demonstrated appreciable alterations compared to the model group. This indicated that loperamide hydrochloride was able to successfully construct a mouse model of constipation, whereas the excipient was ineffective in relieving constipation.

Following probiotic treatment with the BM1, BM2, and PB2 groups, the amount of the faecal water content of the constipated mice increased significantly (*p* < 0.05) compared to the model group, with the effects of BM1 and BM2 being more significant (*p* < 0.01). As for the time to pass the first black stool, all four strains of the probiotics significantly shortened the time to pass the first black stool compared with the model group (*p* < 0.01). The BM1 and BM2 intervention groups took the shortest time to pass the first black stool. The propulsion rate of the small intestine represents the intestinal transit time, and a higher propulsion rate indicates a higher gastrointestinal transport capacity. Compared with the model group, the BM1-treated group showed a significant increase in the small intestinal propulsion rate of constipated mice after intervention with different multi-strain probiotics (*p* < 0.05); although BM2, PB1, and PB2 also tended to increase the small intestinal propulsion rate of constipated mice, there was no significant difference when compared with the model group. In summary, according to the evaluation criteria of the positive results of the “laxative test” in the “Health Food Function Test and Evaluation Method (2022 edition)”, while PB1 could only speed up the intestinal transit time of constipated mice, BM1, BM2, and PB2 showed promising outcomes and could successfully treat the symptoms of constipation.

### 3.4. Different Multi-Strain Probiotics Modulated the Levels of Neurotransmitters and Inflammatory Factors in Mice Serum to Different Degrees

#### 3.4.1. The Effects of Different Probiotic Supplements on Serum Neurotransmitters in Mice

**Figure 4:**
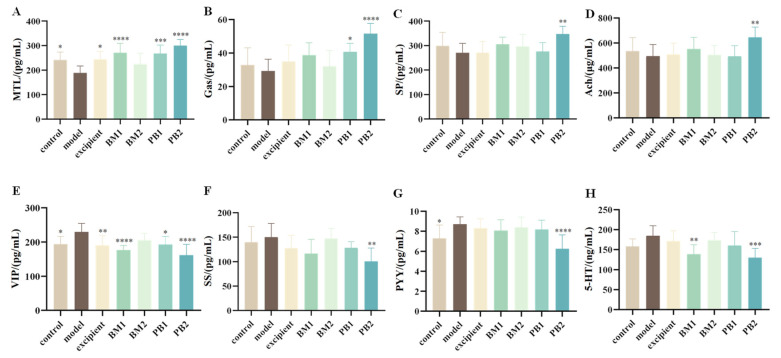
The effects of different probiotic supplements on serum neurotransmitters in mice. (**A**) mouse motilin, MTL; (**B**) gastrin, Gas; (**C**) substance p, SP; (**D**) vasoactive intestinal peptide, VIP; (**E**) somatostatin, SS; (**F**) Peptide YY, PYY; (**G**) acetylcholine, Ach; (**H**) 5-hydroxytryptamine, 5-HT. Data are shown as mean ± standard deviation. One-way ANOVA followed by Fisher’s LSD test for the control and strain groups compared with the model group; *n* = 10, * *p* < 0.05, ** *p* < 0.01, *** *p* < 0.001, **** *p* < 0.0001.

To investigate the effects of different multi-strain probiotics on constipation-related gastrointestinal regulatory transmitters, excitatory (MTL, Gas, SP, and Ach) and inhibitory neurotransmitters (VIP, SS, PYY, and 5-HT) in the serum of constipated mice were evaluated. While Gas, SP, and Ach levels in the blood of model mice treated with loperamide hydrochloride were not substantially altered compared with those in the control group, the levels of the excitatory neurotransmitter MTL were considerably decreased (*p* < 0.05) (Figure 4A–D). Compared with the levels of SS and 5-HT, the serum levels of the inhibitory neurotransmitters VIP and PYY were considerably higher in the mice (*p* < 0.05) (Figure 4E–H). This suggests that constipation triggered by loperamide hydrochloride is associated with abnormal serum levels of MTL, VIP, and PYY. We found that when probiotics were administered, the serum levels of MTL were significantly increased (*p* < 0.001), and VIP levels were significantly downregulated (*p* < 0.05) in the BM1, PB1, and PB2 intervention groups compared with those in the model group and returned to normal levels. By increasing the levels of MTL, VIP, and PYY in the serum, BM1 and PB2 could control the release of digestive neurotransmitters and alleviate constipation.

#### 3.4.2. The Effect of Different Probiotic Supplements on Serum Inflammatory Factors in Mice

**Figure 5:**
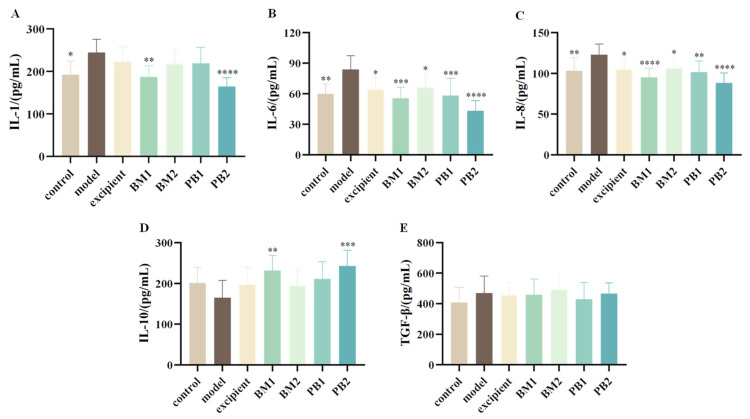
The effects of different probiotic supplements on serum inflammatory factors in mice. (**A**) Interleukin-1, IL-1; (**B**) Interleukin-6, IL-6; (**C**) Interleukin-8, IL-8; (**D**) Interleukin-10, IL-10; and (**E**) transforming growth factor-β, TGF-β. Data are shown as mean ± standard deviation. One-way ANOVA followed by Fisher’s LSD test for the control and strain groups compared with the model group; *n* = 10, * *p* < 0.05, ** *p* < 0.01, *** *p* < 0.001, **** *p* < 0.0001.

The serum levels of pro-inflammatory cytokines (IL-1, IL-6, IL-8) and anti-inflammatory cytokines (IL-10, TGF-β) in mice were assessed in order to quantify the effects of various multi-strain probiotics on the serum levels of inflammatory factors in constipated mice. The serum levels of the pro-inflammatory cytokines IL-1, IL-6, and IL-8 were significantly higher in mice with loperamide hydrochloride-induced constipation than in the control group (*p* < 0.05) (Figure 5A–C). By contrast, there were no statistically significant changes in the levels of the anti-inflammatory cytokines IL-10 and TGF-β (Figure 5D,E). This indicates that the constipation model constructed using loperamide hydrochloride was associated with abnormal levels of the pro-inflammatory cytokines IL-1, IL-6, and IL-8 in the serum. Compared to the model group, all probiotic-treated groups significantly downregulated the serum levels of IL-6 and IL-8 in constipated mice (*p* < 0.05). The BM1 and PB2 treatment groups showed significantly reduced serum levels of IL-1 (*p* < 0.01). In conclusion, BM1, BM2, and PB2 therapies lowered the levels of inflammatory components in the serum and decreased the levels of proinflammatory cytokines in mice with constipation.

### 3.5. Multi-Strain Probiotics Significantly Upregulated the Content of SCFAs in the Faeces of Constipated Mice

**Figure 6:**
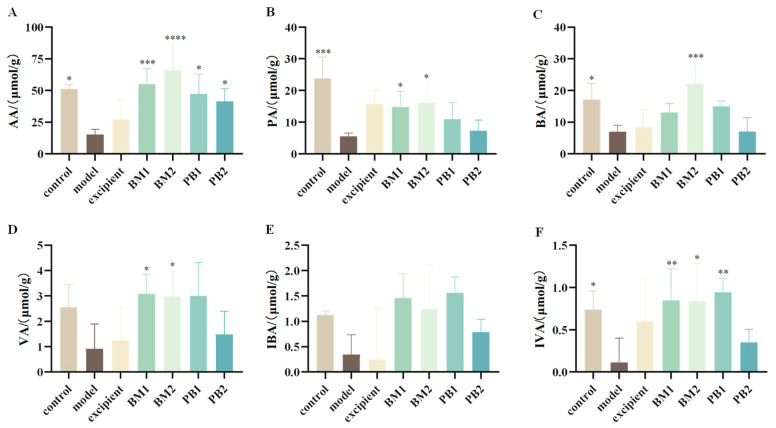
The effects of different probiotic supplements on the content of SCFAs in the faeces of constipated mice. (**A**) acetic acid, AA; (**B**) propionic acid, PA; (**C**) butyric acid, BA; (**D**) valeric acid, VA; (**E**) isobutyric acid, IBA; and (**F**) isovaleric acid, IVA. Data are shown as mean ± standard deviation. One-way ANOVA followed by Fisher’s LSD test for the control and strain groups compared with the model group; *n* = 10, * *p* < 0.05, ** *p* < 0.01, *** *p* < 0.001, **** *p* < 0.0001.

Acetic acid (AA), propionic acid (PA), isobutyric acid (IBA), butyric acid (BA), isovaleric acid (IVA), and valeric acids (VA) were the primary components of SCFAs. Six SCFAs were detected in the faeces of the mice. The levels of AA, PA, BA, and IVA in the faecal SCFAs of mice with loperamide hydrochloride-induced constipation were significantly reduced (*p* < 0.05) (Figure 6A–C,F), whereas the levels of VA and IBA were not statistically different (Figure 6D,E). This indicates that constipation was associated with abnormal levels of SCFAs (AA, PA, BA, and IVA) in mice after loperamide hydrochloride administration. After probiotic intervention, the AA level was significantly higher in all probiotic-treated groups than in the model group (*p* < 0.05), the PA level was significantly increased in the BM1- and BM2-treated groups (*p* < 0.05), and the BA level was significantly upregulated in the BM2-treated group (*p* < 0.001). Moreover, the BM1, BM2, and PB1 treatment groups showed significantly increased IVA content in the faeces of constipated mice (*p* < 0.05). In conclusion, BM1 and BM2 decreased constipation and increased the amount of SCFAs in stools. PB2 only increased the content of acetic acid in the faeces of constipated mice, and SCFAs might not have been the main factor in improving the symptoms of constipation.

### 3.6. Different Multi-Strain Probiotics Improve Constipation Symptoms by Changing the Compositional Structure of Intestinal Flora

**Figure 7:**
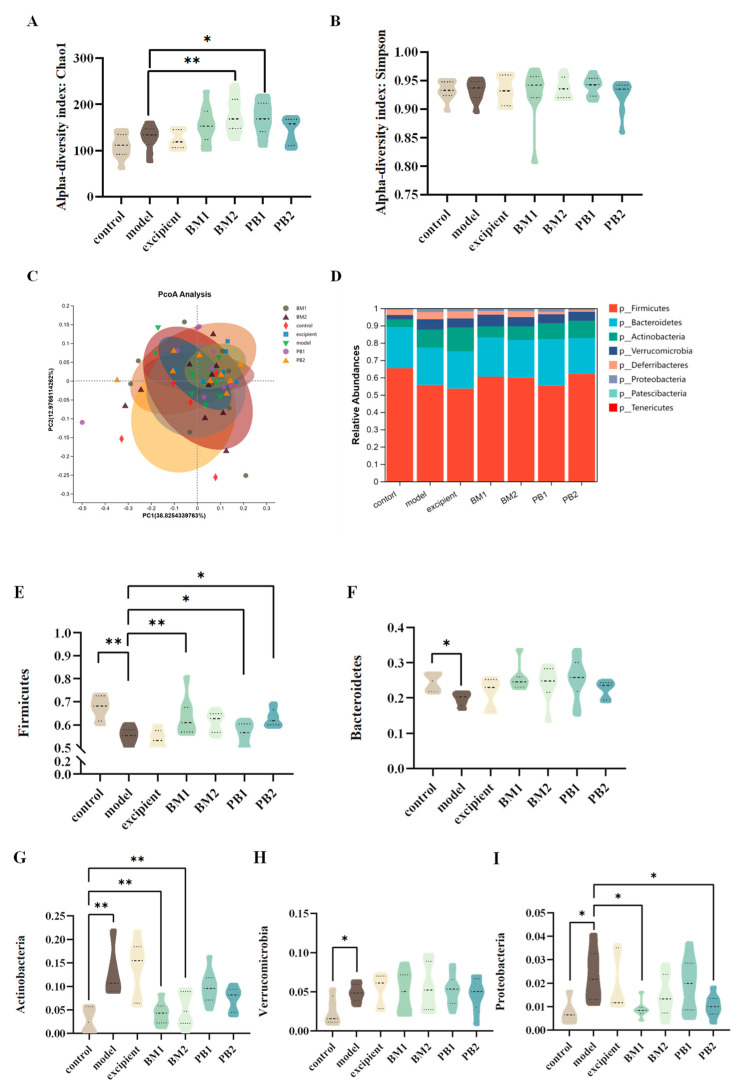
The effects of different probiotic supplements on the diversity and structure of the gut microbiota of constipated mice at the phylum level. (**A**) The Chao1 index of α-diversity; (**B**) The Simpson index of α−diversity; (**C**) An unweighted−UniFrac analysis based on the relative abundance of operational taxonomic units (OTUs) was performed to evaluate β−diversity; (**D**) Phyla of the gut microbiota; (**E**) The Relative abundance of Firmicutes; (**F**) The Relative abundance of Bacteroidetes; (**G**) The Relative abundance of Actinobacteria; (**H**) The Relative abundance of Verrucomicrobia; and (**I**) The Relative abundance of Proteobacteria. Data are shown as mean ± standard deviation. One-way ANOVA followed by Fisher’s LSD test for the control and strain groups compared with the model group; * *p* < 0.05, ** *p* < 0.01.

**Figure 8:**
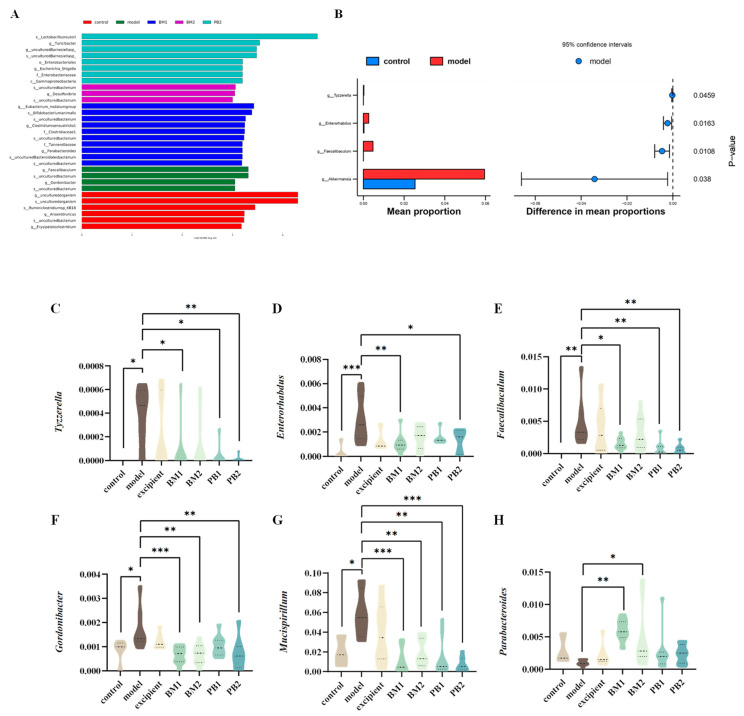
The effects of different probiotic supplements on the gut microbiota of constipated mice. (**A**) Distribution histogram based on LDA (LDA score > 3.0). (**B**) Differential gut microbiota between the model group and control group. The relative abundances of (**C**) *Tyzzerella*, (**D**) *Enterorhabdus*, (**E**) *Faecalibaculum*, (**F**) *Gordonibacter*, (**G**) *Mucispirillum*, and (**H**) *Parabacteroides*. * *p* < 0.05, ** *p* < 0.01, *** *p* < 0.001.

The α-diversity represents the richness and homogeneity of the sample species, while the β-diversity indicates the diversity of species between groups. The α-diversity (Figure 7A,B) and β-diversity (Figure 7C) analyses of the intestinal flora of constipated mice revealed no significant differences in the α-diversity Chao 1 and Simpson indices between the model and control groups. Compared with the model group, the α -diversity Chao 1 index increased significantly (*p* < 0.05) in the BM2 and PB1 intervention groups, whereas there was no statistical difference in the α -diversity Simpson index between the intervention groups. In addition, based on the analysis of β-diversity, the results obtained using PCoA plots showed that the faecal flora of the model group changed significantly (*p* < 0.05) in comparison to that of the control group. This indicates that the overall structure of the intestinal flora in the loperamide hydrochloride-induced constipation model changed. Each bacterial intervention significantly changed the β-diversity of the intestinal flora in constipated mice compared to that of the model group (*p* < 0.05). Further analysis at the phylum level of the intestinal flora (Figure 7D) revealed that the differences between the groups were mainly reflected in the relative abundances of the five microbial phyla: Firmicutes, Bacteroidetes, Actinobacteria, Verrucomicrobia, and Proteobacteria. At the phylum level (Figure 7E–I), the loperamide hydrochloride treatment significantly downregulated the relative abundances of the Firmicute and Bacteroidete microbial phylum (*p* < 0.05). Simultaneously, the relative abundance of the microbial phyla Actinobacteria, Verrucomicrobia, and Proteobacteria was significantly up-regulated (*p* < 0.05). After bacterial intervention, BM1, PB1, and PB2 significantly increased the relative abundance of Firmicutes in the faeces of constipated mice compared with that in the model group (*p* < 0.05), and the relative abundance of Actinobacteria was significantly downregulated in the BM1 and BM2 treatment groups (*p* < 0.01). Moreover, the relative abundance of Proteobacteria was significantly lower in the BM1 and PB2 groups (*p* < 0.05). A further analysis of the microorganisms at the genus level is shown in Figure 8A,B. The characteristic genera of the control group included *Anaerotruncus* and *Erysipelatoclostridium*, and the characteristic genera of the model group included *Faecalibaculum* and *Gordonibacter*. The difference between the model group and control group was mainly observed in the genera *Tyzzerella*, *Enterorhabdus*, *Faecalibaculum*, *Akkermansia*, *Faecalibaculum*, *Gordonibacter*, and *Mucispirillum*, which significantly increased in relative abundance (*p* < 0.05) after loperamide hydrochloride treatment (Figure 8C–G). After bacterial intervention, the relative abundances of *Tyzzerella*, *Enterorhabdus*, *Faecalibaculum*, *Gordonibacter* and *Mucispirillum* in the faeces of mice in the BM1 and PB2 groups were significantly reduced compared to those in the model group (*p* < 0.05). In addition, the relative abundances of *Faecalibaculum*, *Gordonibacter* and *Mucispirillum* in the BM2 treatment group were significantly upregulated (*p* < 0.05). Therefore, probiotics can modify the relative richness of the intestinal flora in mice with constipation and ameliorate intestinal damage caused by loperamide hydrochloride to varying degrees.

### 3.7. Correlation Analysis Showed That Constipation Relief by Different Multi-Strain Probiotics Was Associated with Changes in Intestinal Flora and SCFAs

**Figure 9:**
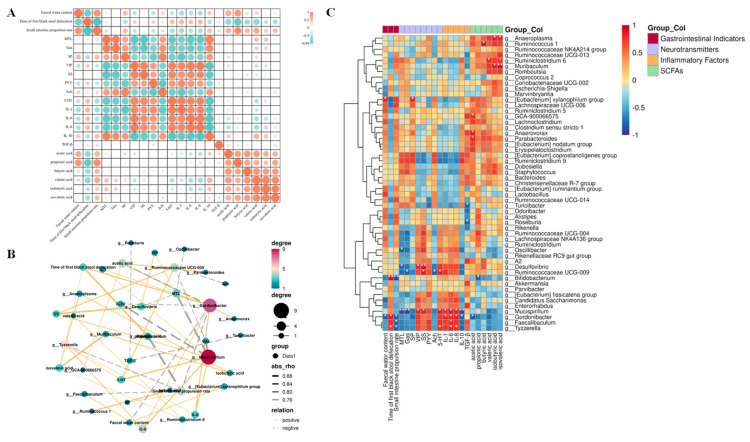
A correlation analysis of intestinal microbiota and physicochemical indices in mice. (**A**) A correlation analysis of gastrointestinal indicators and constipation apparent indices. (**B**) A network diagram of the Pearson correlation analysis. (**C**) A Pearson correlation analysis of the correlation between intestinal bacterial abundance and constipation−related biomarkers. A solid line represents a positive correlation, and a dotted line represents a negative correlation. The thickness of the arrow represents the size of the correlation coefficient. (* *p* < 0.05, ** *p* < 0.01).

The correlation between the apparent indicators of constipation in the gastrointestinal tract and each related test index was established based on the results above, as shown in Figure 9A. The indicators that were positively correlated with stool water content were AA, PA, and SP, while the indicators that were negatively correlated were VIP, IL-1, IL-6, and IL-8, among which PA showed a significant correlation (*p* < 0.05). The indicators that were positively correlated with the time of passing the first black stool were 5-HT, IL-1, IL-6, and IL-8. The indicators that were negatively correlated were AA, PA, BA, VA, and IVA, among which PA showed a significant correlation (*p* < 0.05). Indicators positively correlated with the small intestine propulsion rate included IL-10, AA, PA, BA, VA, and IVA; negatively correlated indicators included 5-HT, IL-1, IL-6, and IL-8, among which PA showed a significant correlation (*p* < 0.05). To further analyse the correlation between the relative abundance of various gut microorganisms at the genus level and constipation-related gastrointestinal indicators, we performed a Pearson correlation analysis. The results shown in Figure 9B,C indicate that the relative abundance of the *[Eubacterium] xylanophilum group* was significantly and positively correlated with faecal water content (*p* < 0.05), whereas the relative abundance of *Gordonibacter* was significantly negatively correlated with faecal water content (*p* < 0.05). The relative abundances of *Bifidobacterium*, *Gordonibacter*, and *Tyzzerella* were significantly and positively correlated with the time to defecate the first black stool (*p* < 0.05). The relative abundances of *Bifidobacterium*, *Gordonibacter*, *Faecalibaculum*, and *Tyzzerella* were significantly and negatively correlated with small intestinal advancement (*p* < 0.05). In addition to this, the relative abundance of *Tyzzerella* also showed a significant positive correlation with the levels of 5-HT, IL-6, and IL-8 (*p* < 0.05); the relative abundance of *Faecalibaculum* showed a significant positive correlation with the level of IL-6 (*p* < 0.05); the relative abundance of *Gordonibacter* was significantly and positively correlated with IL-1, IL-6, and IL-8 levels (*p* < 0.05) and negatively correlated with IL-10 and AA levels (*p* < 0.05). The relative abundance of *Mucispirillum* was significantly and positively correlated with VIP, SS, 5-HT, IL-1, IL-6, and IL-8 contents (*p* < 0.05) and negatively correlated with MTL, Gas, and IL-10 contents (*p* < 0.05); lastly, the relative abundance of *Parabacteroides* was significantly and positively correlated with the content of AA.

In summary, the above results show that BM1, BM2, and PB2 all relieve constipation, but their pathways and advantages are not the same. BM1 and BM2 mainly restored the relative abundances of *Tyzzerella*, *Enterorhabdus*, *Faecalibaculum*, *Gordonibacter*, and *Mucispirillum* in faeces by decreasing the relative abundance of *Parabacteroides*, increasing the level of SCFAs in faeces, restoring MTL and VIP levels, and downregulating the levels of the pro-inflammatory cytokines IL-6 and IL-8 in serum, which in turn repairs the inflammatory response triggered by loperamide hydrochloride and increases the peristalsis of the gastrointestinal tract. PB2, on the other hand, mainly increases the relative abundance of *Tyzzerella*, *Enterorhabdus*, *Faecalibaculum*, *Gordonibacter*, and *Mucispirillum*; increases the level of acetic acid in the stool; upregulates the levels of MTL and SP; downregulates the levels of VIP and PYY; and regulates the levels of the pro-inflammatory cytokines IL-1, IL-6, and IL-8 in the serum. Specific results are shown in Table 4.

## 4. Discussion

Constipation, as one of the common gastrointestinal disorders, is closely related to intestinal flora [37]. According to numerous studies, probiotics help ease constipation by restoring gut flora [38]. Moreover, the effects of single and compound probiotic strains on constipation relief vary, and different combinations of probiotics result in differences in efficacy [1,39,40]. Therefore, this study focused on screening probiotic formulations with constipation-relieving effects and attempted to explain the possible mechanisms of constipation relief.

The results of our study confirmed that all four probiotic formulations met the criteria for positive results in the “Test method for regulating intestinal flora” in the “Health Food Function Test and Evaluation Method (2022 edition)”, and considerably boosted the range of beneficial microorganisms (*Bifidobacterium* and *Lactobacillus*) in the intestinal flora of mice, regulating intestinal flora. Probiotics have been shown in preliminary research to have positive effects on the diseases of the intestinal flora and restore the composition of intestinal flora [41]. Moreover, the four multi-strain probiotics had the potential to alleviate loperamide-induced constipation in mice, and different probiotic formulations resulted in different degrees of improvement in faecal dryness and gastrointestinal motility in constipated mice. After BM1, BM2, and PB2 interventions, the faecal water content of constipated mice, the gastrointestinal transport rate, and intestinal motility increased significantly. Li et al. [42] reported similar findings, which were consistent with our earlier discoveries [31]. BM1 (*B. infantis* BLI-02, *B. animalis* BB-115, and *L. rhamnosus* MP108) performed well in alleviating constipation but contained the least number of strains (with the highest proportion of *Bifidobacteria*). This indicates that the degree of improvement in constipation was not proportional to the number of strains but may be related to the proportion of *Bifidobacteria*. In our previous studies, *Bifidobacterium* was found to have an advantage over *Lactobacillus* in terms of constipation relief [31,35,36], which may be related to the fact that *Bifidobacterium* plays a more reciprocal role in promoting colonization than *Lactobacillus* [43]. *L. paracasei*, one of the most common *Lactobacillus* species, was found to be effective in relieving symptoms of constipation in a combination that contained *L. paracasei* and other strains, with some dose correlation [44,45]. This echoes our results where BM1 is composed of two strains of *Bifidobacterium* and one strain of *Lactobacillus*, whereas BM2 has an additional strain of *L. paracasei*. Our results showed that both formulations were effective in relieving constipation; however, the effect of BM1 was more pronounced, not only increasing the faecal water content of constipated mice and reducing the time it took to pass the first black stool but also significantly increasing the small intestinal propulsion rate of constipated mice. This result also showed that an increase in *Lactobacillus*—but not *Bifidobacterium*—in the probiotic formula for constipation relief did not necessarily increase the effect of constipation relief as was expected.

Constipation alters host gastrointestinal regulatory transmitter levels and inflammatory factor levels, leading to changes in intestinal function [19,46]. By encouraging the formation of pepsin, MTL aids in improving gastrointestinal motility [47]; constipation is accompanied by a decrease in the release of MTL, which reduces gastrointestinal motility. VIP is mostly located in the cells of the digestive tract, where it slows the contraction of the sphincter and smooth muscles, stimulates the pancreas to release bicarbonate, and promotes the secretion of intestinal fluid [48]. PYY, a peptide of 36 amino acids, is an intestinal hormone released by enteroendocrine L cells [49,50]. 5-HT is an important medium for regulating intestinal peristalsis. Qiu et al. [51] found that Lactobacillus saliva Li01 significantly decreased the content of 5-HT in serum and increased the content of 5-HT in the colons of mice with loperamide-induced constipation. Liu et al. [52] found that the secretion levels of gastrointestinal regulatory peptides such as MTL, Gas, ET-1, SS, SP, and VIP in the serum of constipated mice were significantly restored after the administration of a compounded microecological preparation. Our results showed that BM1 and PB2 significantly upregulated MTL levels in the serum of constipated mice and significantly reduced VIP levels, which in turn promoted gastrointestinal motility and small intestinal peristalsis. PB2 also significantly reduced the serum levels of PYY in constipated mice, thus regulating gastrointestinal motility and alleviating the symptoms of reduced gastrointestinal motility caused by loperamide hydrochloride. Previous studies in our laboratory have suggested some strain specificity in the effect of *L. paracasei* on host gastrointestinal regulatory transmitter levels. Wang et al. [53] found that an intraperitoneal injection of PYY inhibited colon advancement in mice. In a study by Liu et al. [54], mice were gavaged with *L. plantarum* CQPC02, a bacterium isolated from Sichuan kimchi, and it was found that LP-CQPC02 intervention increased the levels of MTL, Gas, ET, and Ach and decreased SS levels, which in turn reduced the effects of constipation in mice, maintained intestinal health, and relieved constipation. This is consistent with the finding that BM1 and PB2 relieve constipation, suggesting that the three multi-strain probiotics may relieve constipation by altering the levels of neurotransmitters in the intestinal tract [55], but with slightly different biases. The *Bifidobacterium*-dominated formulation BM1 mainly showed the upregulation of MTL levels in the serum of constipated mice and the downregulation of VIP levels in the serum, promoting intestinal fluid secretion and gastrointestinal motility. The postbiotic PE0401 is a mixture of metabolites from three *Lactobacillus* strains and one *Bifidobacterium* strain. Our results revealed that the addition of PE0401 to BM2—a *Lactobacillus*-dominated formulation of PB2—resulted in increased serum levels of MTL and SP in constipated mice, decreased serum levels of VIP and PYY, and accelerated peristalsis in the small intestine.

Th1 cells, CD4+ cells, macrophages, and dendritic cells generate pro-inflammatory cytokines, which are characterized by the generation of various interleukins (ILs). Constipation has been shown to be associated with inflammation in numerous clinical trials [56]. One investigator evaluated the relationship between inflammatory cytokines and constipation in the elderly and found a significant correlation between the serum levels of inflammatory factors (TNF-α, IL-1, IL-6) and constipation [57]. Our results also showed that constipation was accompanied by inflammation. BM1, BM2, and PB2 significantly reduced the levels of pro-inflammatory cytokines (IL-1, IL-6, and IL-8) in the serum of constipated mice, reducing inflammation while relieving constipation. Shadnoush et al. [58]. evaluated the effectiveness of probiotic yogurt in the treatment of inflammatory bowel disease and found that probiotic yogurt significantly decreased IL-6β and TNF levels and increased IL-8 and IL-10 levels in patients when compared with those that used a plain yogurt treatment.

Euphorbia humifusa-Derived Polysaccharides (EHPs, polysaccharide derivative) can reduce the relative abundance of *Tyzzerella*, alter the composition of the intestinal flora, and alleviate ulcerative colitis (UC) symptoms [59]. *Mucispirillum* is associated with gene expression in mucosal tissues, and alterations in its expression produce an inflammatory response [60]. *Gordonibacter* is an opportunistic pathogenic bacterium, and its increased abundance is linked to the emergence of illnesses, including enteritis and hypertension [61]. By contrast, Parabacteroides can produce succinic acid in the intestine [62]. Tim et al. [63] found statistically significant differences in *Bacteroides fragilis*, *Bacteroides ovatus*, *Bifidobacterium longum*, *Parabacteroides*, and *Alistipes finegoldii* among 76 constipated and healthy children in a comparative analysis of their intestinal flora. *Enterorhabdus* is considered one of the human intestinal pathogens that participates in the fermentation of undigested proteins in the intestinal tract and may destroy intestinal homeostasis. Its products, ammonia and putrescine, are closely related to the occurrence of colorectal cancer [64]. Chen et al. [65] analysed the alterations in saliva microbiota in the normal, small intestinal inflammation and chronic gastritis groups through 16S rRNA gene amplicon sequencing. The levels of *Porphyromonas* and *Faecalibaculum* in gastritis samples were increased, and the enrichments of *Faecalibaculum* and *Kosakonia* in small intestine inflammation samples were elevated compared with those in normal individuals. In this study, we found that the relative abundances of *Tyzzerella*, *Mucispirillum*, and *Gordonibacter* in the faeces of constipated mice were significantly higher, and that of *Parabacteroides* was lower. After bacterial intervention, the relative abundances of *Tyzzerella*, *Mucispirilum*, and *Gordonibacter* in the BM1, BM2, and PB2 groups were significantly decreased, whereas the relative abundance of *Parabacteroides* in the BM1 and BM2 groups was significantly increased in comparison to the state of the groups when quantified before intervention. Moreover, the relative abundances of *Tyzzerella*, *Mucispirillum* and *Gordonibacter* were significantly and positively correlated with IL-6 and IL-8 levels, the relative abundance of *Gordonibacter* was significantly and negatively correlated with acetic acid content, and the relative abundance of *Parabacteroides* was significantly and positively correlated with acetic acid content. In addition, our results showed that propionic acid content was significantly and positively correlated with faecal water content and the small intestinal propulsion rate, and it significantly and negatively correlated with the time of the first black stool evacuation. Similarly, changes in the composition of the intestinal flora affect the content of SCFAs, which are crucial components for maintaining the homeostasis of the intestinal environment [66]. In summary, the *Bifidobacteria*-dominated formulations BM1 and BM2 showed changes in intestinal flora composition and structure; significantly decreased the relative abundance of *Tyzzerella*, *Enterorhabdus*, *Faecalibaculum*, *Gordonibacter*, and *Mucispirillum* in faeces; and increased the relative abundance of *Parabacteroides* in faeces. It also increased the content of SCFAs in faeces, restored the levels of MTL and VIP, and downregulated the levels of the pro-inflammatory cytokines IL-6 and IL-8 in the serum, thus repairing the inflammatory response caused by constipation, promoting peristalsis of the gastrointestinal tract, increasing the water content in faeces, and relieving constipation. *Lactobacillus*-dominated formula PB2 mainly showed a significant decrease in the relative abundance of *Tyzzerella*, *Enterorhabdus*, *Faecalibaculum*, *Gordonibacter*, and *Mucispirillum*; an increase in the level of acetic acid in the stool; upregulation of MTL and SP; downregulation of VIP and PYY; and restoration of inflammatory factor (IL-1, IL-6, IL-8) levels in the serum. Consequently, stool properties changed, and gastrointestinal motility increased.

Constipation relief was not improved with more strains but might have been related to the proportion of *Bifidobacteria* in the formula; the next step is to verify the above speculation through an all-*Bifidobacterial* formula. These results will guide the design of probiotic formulas in the future, not only in terms of the number of strains to include but also in considering the synergistic effect between strains, in order to obtain a low-cost and effective probiotic formula.

## 5. Conclusions

A constipation model was constructed using loperamide hydrochloride to establish the correlation between constipation-related indicators and flora, and the results showed that BM1, BM2, and PB2 could effectively relieve constipation; however, probiotic formulations with different proportions of *Bifidobacterium* and *Lactobacillus* involved different pathways. Among them, the *Bifidobacteria*-dominated formulations BM1 and BM2 mainly showed changes in intestinal flora composition and structure; significantly decreased the relative abundance of *Tyzzerella*, *Enterorhabdus*, *Faecalibaculum*, *Gordonibacter*, and *Mucispirillum*; upregulated the relative abundance of *Parabacteroides*; increased the content of SCFAs in faeces; restored the levels of MTL and VIP; and downregulated the levels of the pro-inflammatory cytokines IL-6 and IL-8 in the serum, thus repairing the inflammatory response caused by constipation. This promoted a peristalsis of the gastrointestinal tract, increasing the water content in faeces and relieving constipation. *Lactobacillus*-dominated formula PB2 significantly decreased the relative abundance of *Tyzzerella*, *Enterorhabdus*, *Faecalibaculum*, *Gordonibacter*, and *Mucispirillum*; increased the level of acetic acid in the stool; upregulated MTL and SP; downregulated VIP and PYY; and restored inflammatory factor (IL-1, IL-6, IL-8) levels in the serum, changing stool properties and increasing gastrointestinal motility.

## Figures and Tables

**Table 1:** List of probiotic formulas.

Group	Containing Strain	Strain Combination
BM1	*B. infantis* BLI-02 *B*. *animalis* BB-115 *L. rhamnosus* MP108	BB-115 + MP108 + BLI-02
BM2	*B. infantis* BLI-02 *B*. *animalis* BB-115 *L. rhamnosus* MP108 *L. paracasei* CCFM2711	BB-115 + MP108 + BLI-02 + CCFM2711
PB1	postbiotic PE0401 *B. infantis* BLI-02 *B*. *animalis* BB-115 *L. rhamnosus* MP108	PE0401 + BB-115 + MP108 + BLI-02
PB2	postbiotic PE0401 *B. infantis* BLI-02 *B*. *animalis* BB-115 *L. rhamnosus* MP108 *L. paracasei* CCFM2711	PE0401 + BB-115 + MP108 + BLI-02 + CCFM2711

**Table 2:** List of animal testing groups.

Group	Gavage Substance	Gavage Concentration	Gavage Volume
control	normal saline	-	0.2 mL
model	loperamide hydrochloride	15 mg/(kg·bw)	0.2 mL
excipient	maltodextrin	500 mg/(kg·bw)	0.2 mL
BM1	BB-115 + MP108 + BLI-02	4.1 × 10^8^ CFU/g	0.2 mL
BM2	BB-115 + MP108 + BLI-02 + CCFM2711	4.1 × 10^8^ CFU/g	0.2 mL
PB1	PE0401 + BB-115 + MP108 + BLI-02	4.1 × 10^8^ CFU/g	0.2 mL
PB2	PE0401 + BB-115 + MP108 + BLI-02 + CCFM2711	4.1 × 10^8^ CFU/g	0.2 mL

**Table 3:** The effect of different probiotic supplements on the body weight of mice (g, $\bar{x}$ ± SD).

Group	Initial Weight	Final Weight	*p*
control	23.16 ± 1.06	25.04 ± 1.66	-
model	23.99 ± 1.52	26.14 ± 2.29	<0.05
excipient	24.44 ± 1.17	25.78 ± 1.61	-
BM1	22.89 ± 0.79	24.74 ± 1.19	-
BM2	23.86 ± 1.05	26.07 ± 1.21	<0.05
PB1	24.77 ± 1.61	26.81 ± 1.15	-
PB2	22.82 ± 0.65	24.49 ± 1.08	-

**Table 4:** The summary of this study.

Factors		Model	BM1	BM2	PB2	Figures
***Gastrointestinal Indices***	Faecal water content	↓	↑	↑	↑	Figure 3A
	Time of first black stool defecation	↑	↓	↓	↓	Figure 3B
	Small intestine propulsion rate	↓	↑	-	-	Figure 3C
***Neurotransmitters***		MTL↓, VIP↑, PYY↑	MTL↑, VIP↓	-	MTL↑, VIP↓, PYY↓	Figure 4
***Inflammatory*** ***Factors***		IL-1↑, IL-6↑, IL-8↑	IL-1↓, IL-6↓, IL-8↓	IL-6↓, IL-8↓	IL-1↓, IL-6↓, IL-8↓	Figure 5
***SCFAs***		AA↓, PA↓, BA↓, IVA↓	AA↑, PA↑, IVA↑	AA↑, PA↑, BA↑, IVA↑	AA↑	Figure 6A
***Intestinal Flora***		*Tyzzerella*↑, *Enterorhabdus*↑, *Faecalibaculum*↑, *Gordonibacter*↑, *Mucispirillum*↑	*Parabacteroides*↑, *Tyzzerella*↓, *Enterorhabdus*↓, *Faecalibaculum*↓, *Gordonibacter*↓, *Mucispirillum*↓	*Parabacteroides*↑, *Gordonibacter*↓, *Mucispirillum*↓	*Tyzzerella*↓, *Enterorhabdus*↓, *Faecalibaculum*↓, *Gordonibacter*↓, *Mucispirillum*↓	Figure 7 and Figure 8

Model: Compared with the control group, the corresponding index significantly increased by ↑, the corresponding index decreased significantly by ↓. Others: Compared with the model group, the corresponding index significantly increased by ↑, the corresponding index decreased significantly by ↓.

## Data Availability

The datasets generated and analysed during this study are available from the corresponding author on reasonable request.
